# Cost-effectiveness of left atrial appendage occlusion during cardiac surgery in France: An economic evaluation based on the LAAOS III study

**DOI:** 10.1371/journal.pone.0302517

**Published:** 2024-05-09

**Authors:** Manon Benmalek, Martin Connock, Léa Savio, Jean-François Obadia, Xavier Armoiry

**Affiliations:** 1 Pharmacy Department, Edouard Herriot Hospital, Hospices Civils de Lyon, Lyon, France; 2 Warwick Medical School, University of Warwick, Coventry, United Kingdom; 3 Hôpital Cardiovasculaire Louis Pradel, Chirurgie Cardio-Vasculaire et Transplantation Cardiaque, Hospices Civils de Lyon and Claude Bernard University, Lyon, France; 4 School of Pharmacy (ISPB), UMR CNRS 5510 MATEIS, University of Lyon, Lyon, France; BSMMU: Bangabandhu Sheikh Mujib Medical University, BANGLADESH

## Abstract

**Objectives:**

Left atrial appendage occlusion during cardiac surgery is a therapeutic option for stroke prevention in patients with atrial fibrillation. The effectiveness and safety of left atrial appendage occlusion have been evaluated in several studies, including the LAAOS-III trial. While these studies have demonstrated efficacy and safety, the long-term economic impact of this surgical technique has not yet been assessed. Here, we aimed to evaluate the cost-effectiveness and cost-utility of left atrial appendage occlusion during cardiac surgery over a long-term time horizon.

**Methods:**

Our study was based on a model representing an hypothetical cohort with the same characteristics as LAAOS-III trial patients. We modelled the incidence of ischemic strokes and systemic embolisms in each intervention arm: "occlusion" and "no-occlusion," using a one-month cycle length with a 20-year time horizon. Regarding occlusion devices, sutures, staples, or an approved surgical occlusion device (AtriClip^™^—AtriCure, Ohio, USA) could be used.

**Results:**

Our model generated an average cost savings of 607 euros per patient and an incremental gain of 0.062 quality-adjusted life years (QALYs), resulting an incremental cost-utility ratio (ICUR) of €-9,775/QALY. The scenario analysis in which occlusion was systematically performed using the AtriClip^™^ device generated an ICUR of €3,952/QALY gained.

**Conclusions:**

In the base-case analysis, the strategy proved to be more effective and less costly, confirming left atrial appendage occlusion during cardiac surgery as an economically dominant strategy. The scenario analysis also appeared cost-effective, although it did not result in cost savings. This study provides a new perspective on the assessment of the cost-effectiveness of these techniques.

## 1. Introduction

Atrial fibrillation (AF) is the most common form of sustained cardiac arrhythmia in adults [1] and is an independent risk factor for stroke, resulting in an approximate 3- to 5-fold excess risk [2] compared to patients who do no present AF.

The left atrial appendage (LAA) is considered to be a preferred site of thrombus formation in non-valvular AF [3] and can be a source of embolic strokes, which has led to proposed adjunctive procedures of surgical LAA closure during open-heart surgery. Until recently, the level of evidence supporting surgical LAA occlusion was limited in the absence of data based on large randomised controlled trials (RCT) [4].

Since the release of 2020 European Society of Cardiology (ESC) and European Association of Cardio-Thoracic Surgery (EACTS) guidelines, the results of the Left Atrial Appendage Occlusion Study (LAAOS) III trial have become available [5]. This large RCT included more than 4700 patients from 105 centers in 27 countries and demonstrated that in patients with AF undergoing cardiac surgery with concomitant LAA occlusion the risk of stroke or systemic embolism was reduced by 33% when compared to patients who did not receive concomitant occlusion. As part of a secondary objectives of the trial, a cost analysis separately published by Eqbal et al. [6] showed that, from the perspective of the Centers for Medicare and Medicaid Services, concomitant surgical LAA was an economically dominant strategy taking into account medical direct costs recorded during an observation of 3.8 years in average.

Despite these encouraging results, the long-term economic impact of LAA occlusion during cardiac surgery has yet to be estimated. Similarly, the impact of surgical concomitant LAA occlusion on quality-adjusted life expectancy has not previously been reported.

Here, we aimed to evaluate the cost-effectiveness and cost-utility of LAA Occlusion during Cardiac Surgery over a lifetime horizon hypothesizing that LAA occlusion would represent a cost-effective or cost-saving option compared to standard of care.

This report has followed the Consolidated Health Economic Evaluation Reporting Standards (CHEERS) guidelines [7].

## 2. Materials and methods

### Overview

We evaluated the cost-effectiveness and cost-utility of Left Atrial Appendage Occlusion during cardiac surgery (LAAOs) versus cardiac surgery alone, based on published results of LAAOS III.

The cost-effectiveness was assessed as a cost per event avoided taking into account any stroke (except haemorrhagic stroke) and/ or non-cerebral systemic embolism.

An economic evaluation assessed as a cost per life-year gained was deemed futile in the absence of difference of life expectancy between both strategies as suggested by the main results of the LAAOS III trial.

However, a difference in terms of events avoided could be associated with differences in terms of quality of life (QoL) which has justified a cost-utility analysis as assessed by a cost per QALY gained (quality-adjusted life year).

While LAAOS III investigators conducted an economic evaluation limited to the observation period of patients in the trial (ie 3.8 years in average), [6] we considered a lifetime horizon up to 20 years. To this end, we modelled lifelong costs and clinical outcomes including health-related quality of life (HRQoL).

We adopted the French social security perspective assuming that some LAAOs devices may be covered through specific funding.

### Population and compared strategies

The population was a cohort with the same characteristics to patients in the study LAAOS III [5].

This trial enrolled 4,770 patients (history of atrial fibrillation, score CHA₂DS₂ -VASc ≥ 2, mean age: 71 years), with 2,379 in the “occlusion” group and 2391 in the “no occlusion” group.

We compared the two following strategies: LAAO during cardiac surgery (LAAOs) versus cardiac surgery alone in patients with a history of atrial fibrillation undergoing cardiac surgery for another indication.

In the LAAOS III study [5], left atrial appendage occlusion was performed with the use of any of the following techniques: amputation and closure (preferred), stapler closure, double-layer linear closure from within the atrium, or closure with an approved surgical occlusion device. As of November 2023, the only medical device labelled with CE marking for LAA occlusion during cardiac surgery is the AtriClip^™^ (AtriCure, Mason, Ohio, USA). In France, this device is covered through Diagnosis-related groups (DRG) ‘s funding.

### Description of the model and assumptions

**Fig 1:**
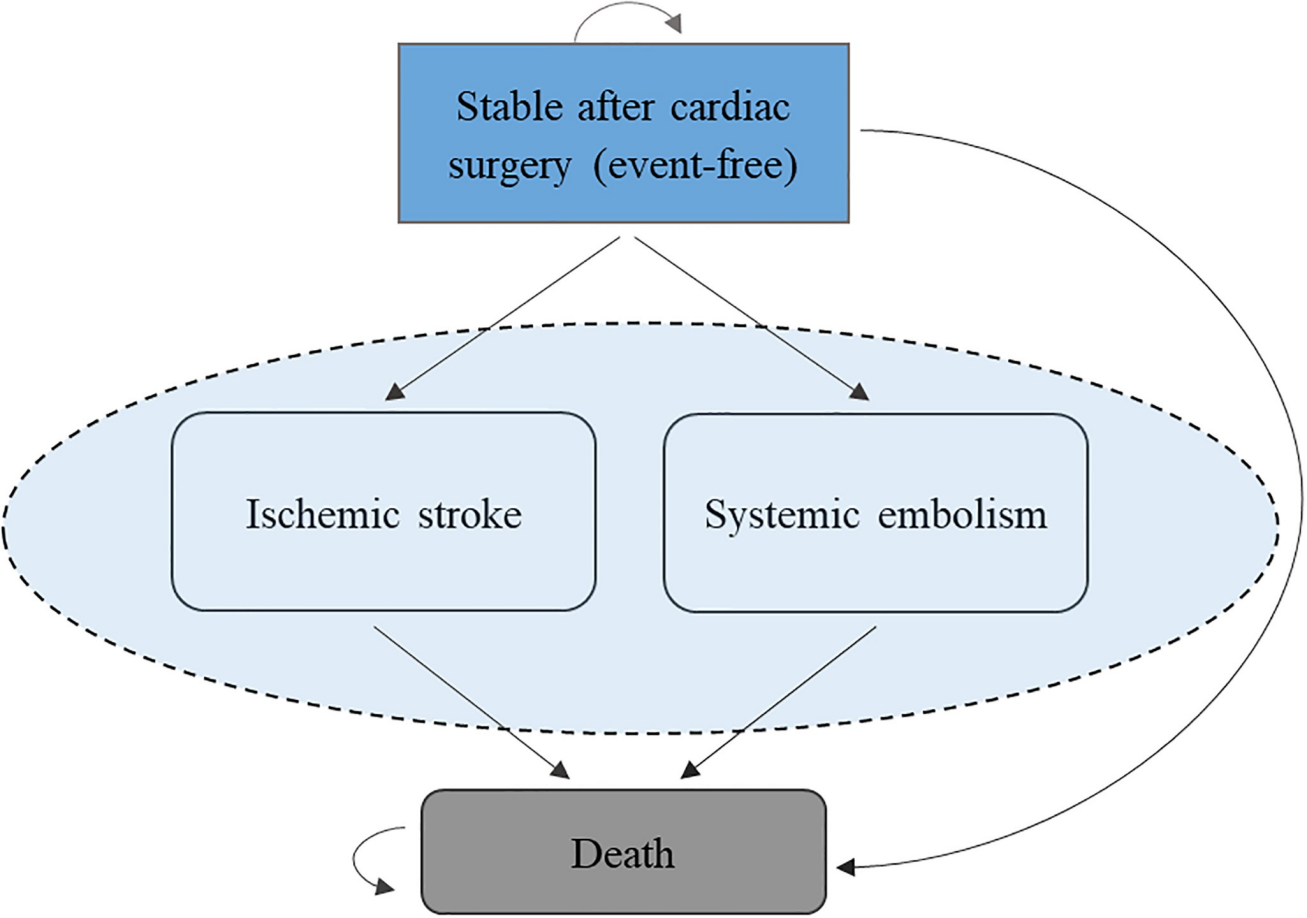
Illustrative structure of the model.

We developed a partitioned health state model (Fig 1) with four mutually exclusive health states: patient alive free of ischemic stroke (IS) or systemic embolic (SE) event, patient alive with at least one IS event, patient alive with at least one SE event, and dead.

The transition probabilities between health states were derived based on results from the LAAOS III study [5]. A cycle length of one month was selected; each month was associated with a cost and a utility value for each health state so that monthly costs and quality-adjusted months gradually accumulated within each arm to the time horizon. In the structure of the model, the occurrence of events generated loss of utility (disutility) and associated costs. Similarly, we applied monthly disutility resulting from patients’ aging.

The model outputs allowed the calculation of incremental difference in costs and consequences, either expressed as quality-adjusted life months/years (cost-utility) or number of events (cost-effectiveness).

Owing to the constraints of data reported from the trial [5, 6], we have made the following assumptions:

- We assumed that the number of IS events equalled the number of patients who had at least one event, and that patients that experienced SE had a total of two events per patient; assumptions that are consistent with results reported by Eqbal et al. [6], based on the same source.
- There was no detailed information on the cumulative incidence of stroke or systemic embolism event separately, we therefore assume each event trajectory to follow that of the combined curve as reported by Whitlock et al. [5].
- We calculated weighted costs and disutilities for IS and SE based on the distribution of event severity (minor, moderate, severe, and fatal) as reported by *Eqbal et al* [6].
- The rate of all- cause death was much higher than the rate of main event measured in the trial, therefore the model assumed a certain rate of background mortality as described in the modelling section.
- We modelled the disutility and costs related to other major cardiovascular events based on rates observed in the trial for other stroke, myocardial infarction, bleeding event, and hospitalisation for heart failure.
- We did not differentiate stroke and systemic embolism according to the timing of occurrence after cardiac surgery (ie. < 30 days or >30 days).

### Time-to event parametric modelling

#### Mortality

The LAAOS III RCT [5] identified the number of deaths from any cause recorded in each arm over the observed follow up as: 538 in 2379 patients in the occlusion arm, and 537 in 2391 patients in the no-occlusion arm. Since there was clearly no significant difference in all-cause mortality between the two arms, we estimated the proportion remaining alive in each arm to be 100% minus 22.54%. Mean follow up was 3.8 years; we therefore adopted the assumption that survival at 3.8 years was 77.46%. There were many study centres in LAAOS III; how French patients of equivalent status would have survived with or without occlusion is unknown. Since the economic model aimed at a French HTA perspective we estimated the survival of a French general population matched by mean age and gender mix to that in LAAOS III population. S1 Fig shows the resulting survival estimate and indicates that a Gompertz parametric model provides a good fit to the estimate. To obtain an estimate of a LAAOS III French population we determined the Gompertz scale parameter required to generate 77.46% survivors at 3.8 years while retaining the shape parameter for a mean age and gender matched French population (S2 Fig). The plausibility of overall survival (OS) estimates was consistent with data in the literature [8, 9].

#### Cumulative events

The LAAOS III RCT reported Kaplan-Meier plots for the cumulative incidence of IS or SE over a total follow-up period of 75 months. The cumulative total number of these events for patients without occlusion (n = 2391) was 168 IS and 7 SE, while patients whose atrial appendage was occluded (n = 2379) the total events were 109 IS and 6 SE. Thus, strokes were by far the dominant events and occlusion reduced their incidence considerably. Whitlock and al. did not publish separate KM plots for each of the two event types. The composite KM plots predominantly reflect stroke. An adjusted hazard ratio between arms was estimated to be 0.67 (95% CI: 0.53–0.85).

Our reconstructed KM plots for cumulative incidence of stroke or embolism are shown in S3 Fig.

The initial rate of accumulation over the first few months is rapid and this rate has declined by about six months and is followed by a slowly increasing rate of accumulation particularly in the non-occlusion arm.

To model cumulative incidence, we fit parametric models separately to each arm; such models allow extrapolation over an extended time horizon beyond the observation period. According to information criteria the best performing model for both arms was the bathtub model (S1 Table). The hazard versus time of bathtub models [10] is “U” shaped and is described by three parameters: hazard = β * t + (γ / (1+ α * t))

**Fig 2:**
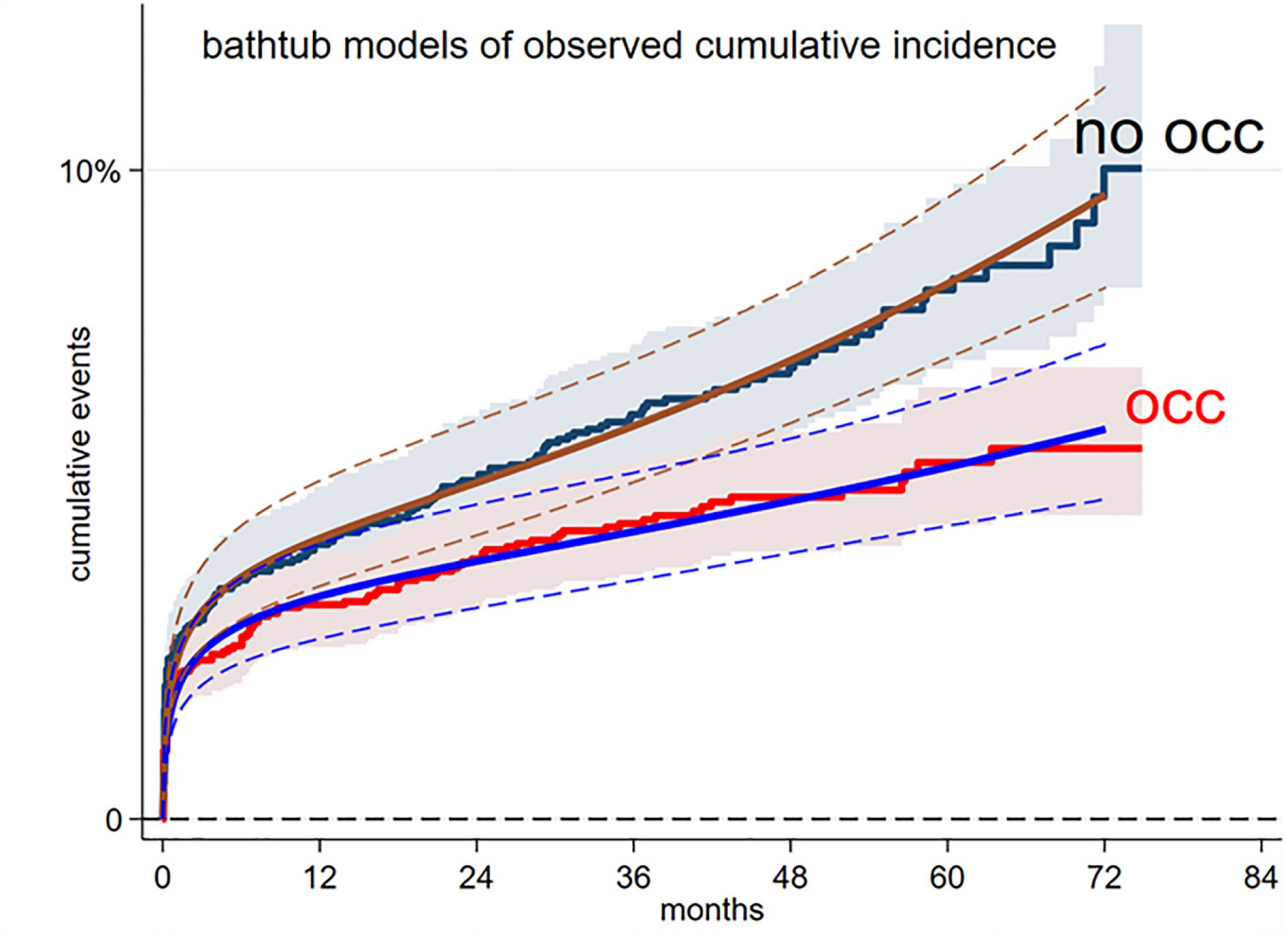
Reconstructed Kaplan-Meier plots for cumulative incidence of events and bathtub models with 95% CI.

This allows for rapid of initial accumulation of events at a rate that gradually declines, followed later by a gradually increasing hazard and increasing rate of accumulation of events. The resulting bathtub models are shown in Fig 2.

**Fig 3:**
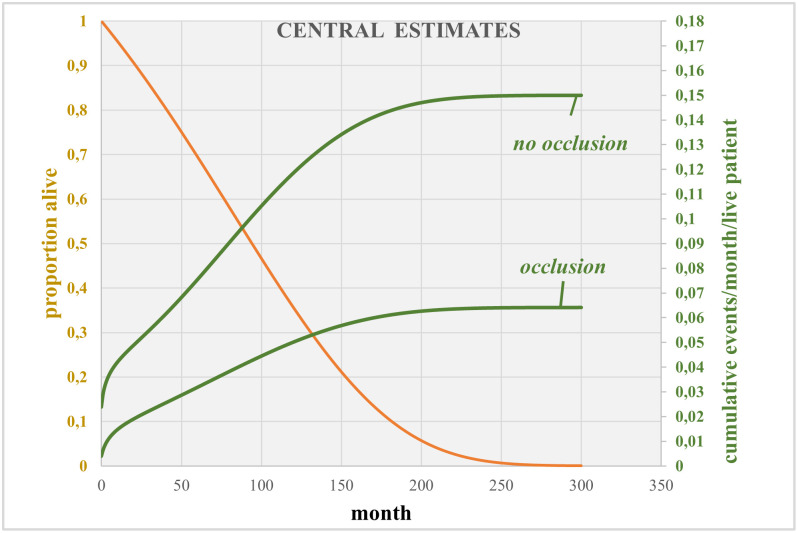
Bathtub models of accumulation of events (stroke or embolism) in each arm of LAAOs III.

The bathtub model for the non-occlusion arm provides a good fit particularly over the first post-operative months when events are most common. During this critical period the bathtub model for the occlusion arm tends to over-estimate events relative to that observed. For this reason, in the economic model, we used the observed accumulation of events to 75 months and the bathtub model for extrapolation (S4 Fig). The parametric model allows calculation of the number of new events each month (cumulative events at time *t1* months minus cumulative vents at time *t1-1*). The bathtub models predict increasing accumulation of stroke or embolism events through time and beyond when patient numbers are becoming low due to mortality (S4 Fig). We therefore estimated the “events / month / live patient” and thence the cumulative “events / month / live patient” for each arm. The results from the bathtub models are shown in Fig 3.

### Model inputs

#### Costs

We have included direct costs, whether medical or non-medical (Table 1).

**Table 1:** Costs and utilities inputs, and main settings of the model.

Parameters	Base case	Sensitivity analysis	Source for the base case
**Costs**			
wClosure technique	192 €	+/- 20%	
Index hospitalization	23,347 €		ENCC 2021
Stable disease post-surgery	142€/month		2018 Le Heuzey [11]
Cost IS in occlusion group from day 0 to 90	12,798 €		2013 Chevreul [12] and 2020 Barral [13], with mRS rate of LAAOS III [5]
Cost IS in occlusion group from day 90 to 1year	2,559 €		
Cost IS in occlusion group after 1 year (per year)	1,926 €		
Cost IS in non-occlusion group from day 0 to 90	13,089 €		
Cost IS in non-occlusion group from day 90 to 1year	2,732 €		
Cost IS in non-occlusion group after 1 year (per year)	2,075 €		
Cost HS in occlusion group from day 0 to 90	12,184 €		2014 Lanitis [14], with mRS rate of LAAOS III
Cost HS in occlusion group from day 90 to 1year	2,718 €		2020 Barral [13], with mRS rate of LAAOS III
Cost HS in occlusion group after 1 year (per year)	2,062 €		
Cost HS in non-occlusion group from day 90 to 1year	10,041 €		2014 Lanitis [14], with mRS rate of LAAOS III
Cost HS in non-occlusion group from day 0 to 90	2,608 €		2020 Barral [13], with mRS rate of LAAOS III
Cost HS in non-occlusion group after 1 year (per year)	1,967 €		
Cost major bleeding (1 month)	6,841 €		2016 Cotte [15]
Cost MI (from day 0 to 90)	5,286 €		2014 Lanitis [14]
Cost post MI per month	429 €		
Cost SE (from day 0 to 90)	3,373 €		
Cost post SE per month	245 €		
Cost HF hospitalization	5,101 €		ENCC 2021
**Utilities**			
Stable disease post-surgery	0,794	+/- 20%	2023 Lyth [16]
Disutility age per month	-0,001		
IS disutility in occlusion group from day 0 to 30	-0,3196		2013 Luengo-Fernandez [17], with mRS rate of LAAOS III
IS disutility in occlusion group from month 1 to 12	-0,2281		
IS disutility in occlusion group after 1 year	-0,2247		
IS disutility in non-occlusion group from day 0 to 30	-0,3836		
IS disutility in non-occlusion group from month 1 to 12	-0,2886		
IS disutility in non-occlusion group after 1 year	-0,2596		
HS disutility from month 0 to 6	-0,27		2023 Lyth [16]
HS disutility from month 6 to 12	-0,20		
HS disutility from month 12 to 24	-0,18		
HS disutility after 24 months	-0,04		
Major bleeding disutility (1 month)	-0,0199		2016 Kaier [18]
MI disutility (from day 0 to 90)	-0,100		2021 Walter [19]
Post MI disutility (lifelong)	-0,060		
SE disutility (lifelong)	-0,100		2014 Lanitis [14]
HF hospitalization disutility (apply only one month)	-0,100		2017 Pufulete [20]
Discount rate	2.50%	0% to 5%	French guidelines [21]
OS model	Gompertz distribution	95%CI	Estimation from LAAOS III study [5]
Cumulative Event/month	Bathtub model	+/- 20%	Estimation from LAAOS III study [5]

CI, confidence interval; ENCC, Étude nationale de coûts à méthodologie commune; HF, heart failure; HS, hemorrhagic stroke; IS, Ischemic stroke; LAAOS, Left atrial appendage occlusion study; MI, myocardial infraction; OS, overall survival; SE, systemic embolism; wClosure technique: cost adjusted with percentages of occlusion device, staple and suture used.

The following healthcare resource utilisation was accounted for: 1)- Index hospital stay for cardiac surgery, including or excluding the use of occlusion medical device; In the base case, we considered the distribution of occlusion techniques used in the LAAOS III trial: AtriClip^™^: 15.1%, stapler: 11.2%, sutures: 69,5%; we undertook sensitivity analyses to take into account the variation in costs of occlusion techniques; 2)- Hospitalisation for stroke/systemic embolism; 3)- Consequences resulting from stroke/systemic embolism (including medication, hospital/ambulatory care, medical transport).

Other expenses, corresponding to events occurring during the course of follow-up [5], were estimated based on information reported by Eqbal et al.: Hospitalisation for heart failure, Major bleeding event, Haemorrhagic stroke, and Myocardial infarction.

These resources were valued in Euros according to tariffs observed during Year 2023.

The sources of costs were as following: hospital cost Database (PMSI, ENC) / literature search for costs of event with no official fare.

#### Effectiveness and utility inputs

For the effectiveness, we considered the number of events avoided, the event being described as *the first occurrence of ischemic stroke (including transient ischemic attack with positive neuroimaging) or noncerebral systemic embolism during follow-up* [5].

To take into account the number of events, we relied on data extrapolated up to 20 years (see modelling section).

For utility inputs, in the absence of published data relevant to the trial, we undertook a non-systematic literature review to search for estimates relevant to events occurring during patients’ follow-up (Table 1).

### Base- case, scenario, and sensitivity analyses

We calculated the incremental cost-effectiveness ratio (ICER) as monetary costs (€) per embolic event avoided, and the Incremental Cost-Utility Ratio (ICUR) as monetary costs (€) per QALY gained.

We discounted future costs and benefits at a rate of 2.5%/year as per the French recommendations [21]. The rate of discounting was included in sensitivity analyses.

Our base-case analyses relied on data from the LAAOS III study, with results extrapolated to a lifetime horizon, and using the distribution of LAA closure technique as described by Whitlock and al. [5]

In a scenario analysis (SA), we considered the use of the Atriclip^™^ device in 100% of occlusions assuming that an additional cost to DRG’s funding would be granted to cover the cost of the device.

In both the base-case and SA, we assumed no difference in survival outcomes between the two arms.

We undertook one-way deterministic sensitivity analyses for all parameters in order to assess the impact that a fixed change in each parameter has on the ICER. The sensitivity analysis considered “optimistic” or “pessimistic” assumptions which mainly consisted in applying +/-20% to the model inputs. These were displayed in Tornado diagrams.

For multivariate sensitivity analyses, we variously modified the most influential model inputs identified in univariate sensitivity analysis to generate pairs of costs (euros) and benefit (QALYs) for each arm. These were then used in bootstrapping [22] with 1000 iterations to generate the joint distributions of incremental costs and QALYs. The results were graphed using the Stata ellip package [23].

### Ethics statement

This study used data available in the public domain, therefore it did not require an ethics approval or written or verbal consent.

## 3. Results

The survival model predicted an undiscounted life expectancy of 8.3 years which applied to both strategies. Other survival estimates were: 5 year, 10 year, 15 year, and 20 year- survival rates of 69%, 35%, 10% and 1% respectively; median OS was estimated at 7.8 years.

The discounted life-expectancy was calculated at 7.2 years.

### Base case

Our model generated an undiscounted lifetime number of events per patient of 0.081 in the occlusion group and 0.153 in the no-occlusion group, which resulted in an incremental 0.072 event avoided per patient.

After discounting, the numbers of event avoided per patient in the occlusion and no-occlusion groups were 0.073 and 0.133 respectively (0.06073 event avoided per patient).

Discounted total QALY in the occlusion and no-occlusion was respectively 5.20 and 5.14 in the no-occlusion group, resulting in an incremental 0.062 QALY gain.

Discounted total costs were €39,522 in the occlusion group and €40,129 in the no-occlusion group (incremental cost of—€607).

These results generated a base case discounted ICER of €-9,998/event avoided and ICUR of € -9,775/ QALY gained suggesting economic dominance.

**Fig 4:**
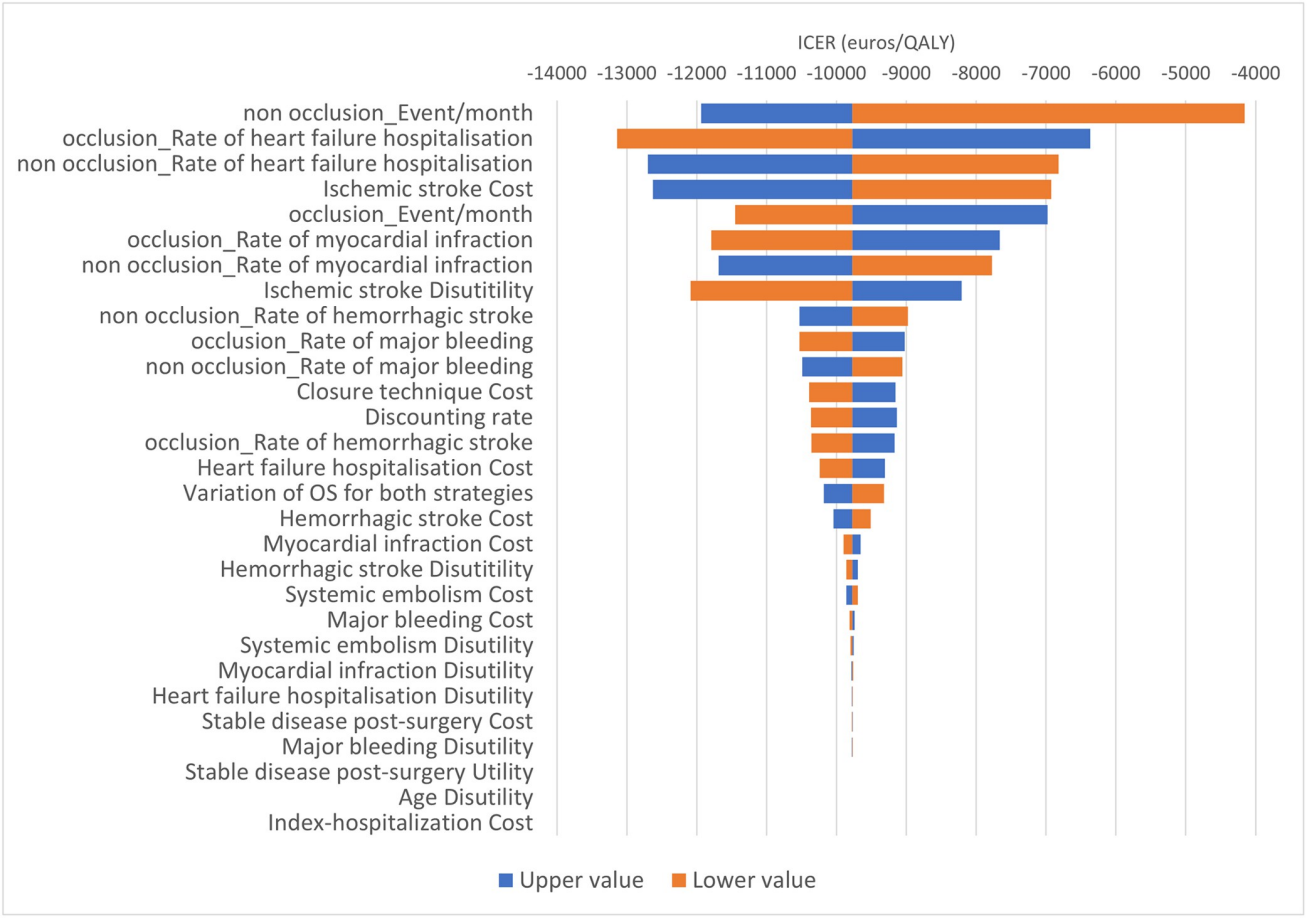
One-way sensitivity analyses: Tornado diagram. Effect of parameter variation on the incremental cost-utility ratio. Orange bars represents the lower bound, blue bars represents the upper bounds. *OS*, *overall survival*.

One-way sensitivity analyses (Fig 4) showed the most influential parameters on the ICUR were: event rate per month (IS and SE) in the “non occlusion” and “occlusion" group, cost/utility of IS, and rates of myocardial infarction and hospitalization for heart failure.

Based on the most influential variables, we performed a multivariate sensitivity analysis taking into account the four following variables: event rate per month in the “non occlusion” and “occlusion" group; cost of IS; utility of IS; and OS.

We decided not to include variations on hospitalization for heart failure and myocardial infarction because these were not statistically different in the LAAOS III trial [5].

**Fig 5:**
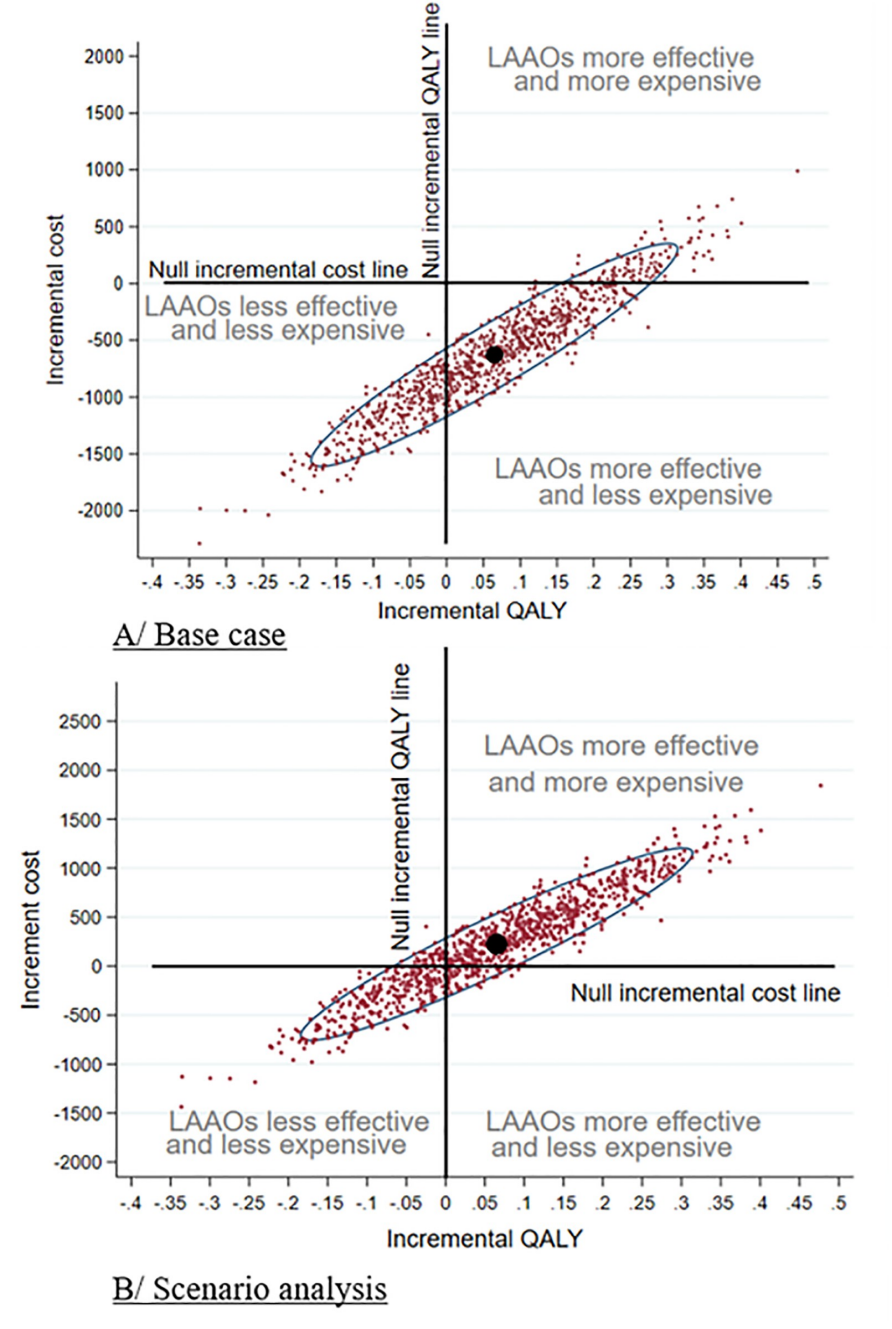
Multivariate analysis showing the distribution of 1000 mean-centred replicates in tne cost-effectiveness plane; A) Base case analysis (occlusion using methods described in LAAOSIII). B) Scenario analysis (occlusion using the AtriClip^™^ device) -.Ellipses represent 95% CI for the 1000 means-centred replicates.

**Fig 6:**
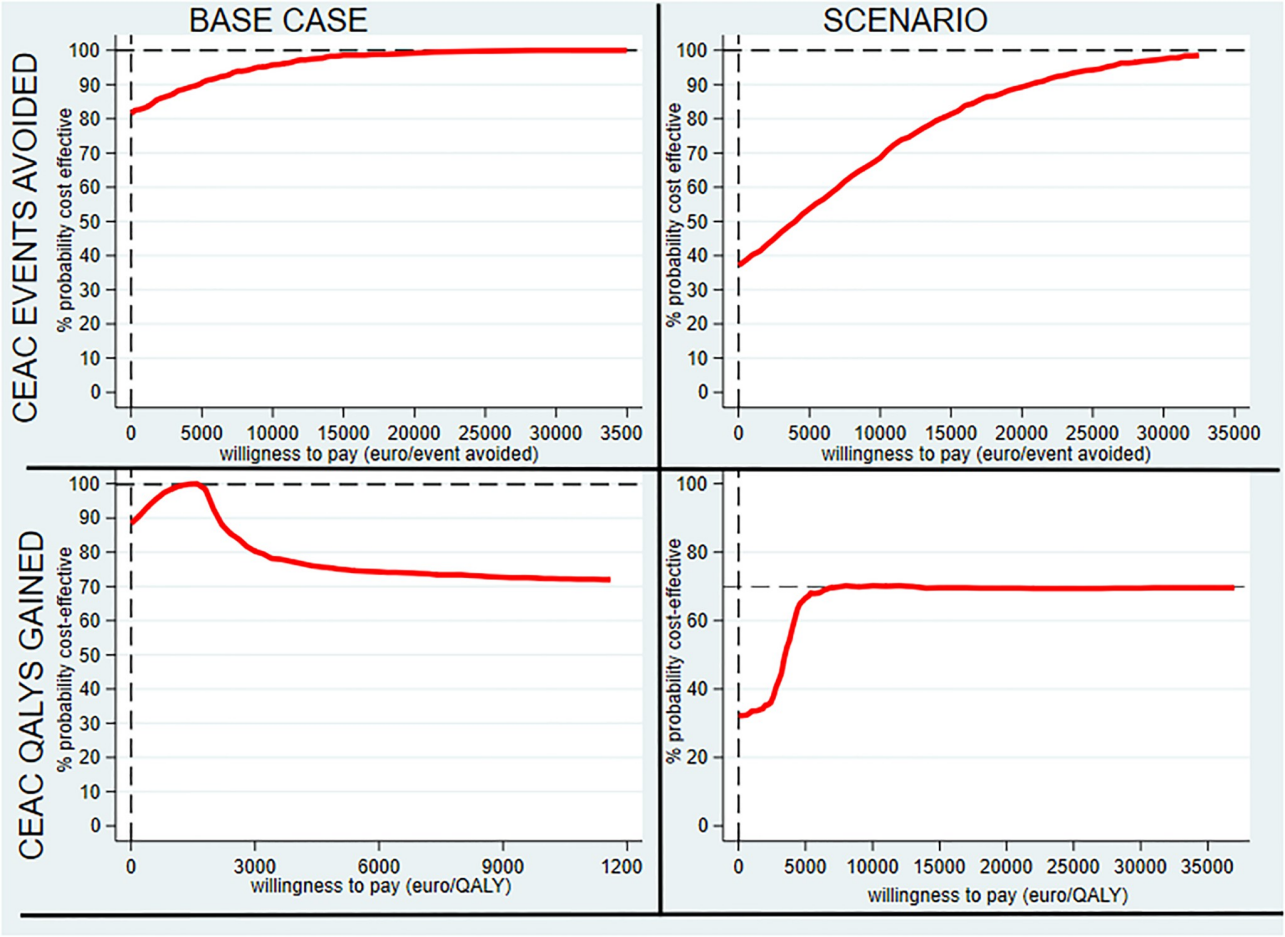
Cost-effectiveness acceptability curve (CEAC); Left: for base case analysis. Right: for scenario analysis.

Fig 5A shows that majority of the replicates (58.5%) were located in the south-east quadrant (denoting an economic dominance for occlusion). The replicates also extended to the south-west quadrant (30.5%) where occlusion is less expensive but less effective, and the north-east quadrant (11%) where occlusion is more effective but more expensive. At a willingness-to-pay (WTP) threshold of €30,000/QALY, occlusion had 78% of chance of being cost-effective (Fig 6).

For the cost/event analysis, we used same variables excepting utility of IS. The S5A Fig) shows that majority of the replicates are located in the southeast quadrant of the CE plane and there are some replicates extend into the northeast quadrant.

### Scenario analyses

Under scenario analysis (SA), discounted total costs were €40,375 in the occlusion group and €40,129 in the no-occlusion group (incremental cost of +€246).

These results generated a discounted ICER of €4,042/event avoided and ICUR of €3,952/ QALY gained.

**Fig 7:**
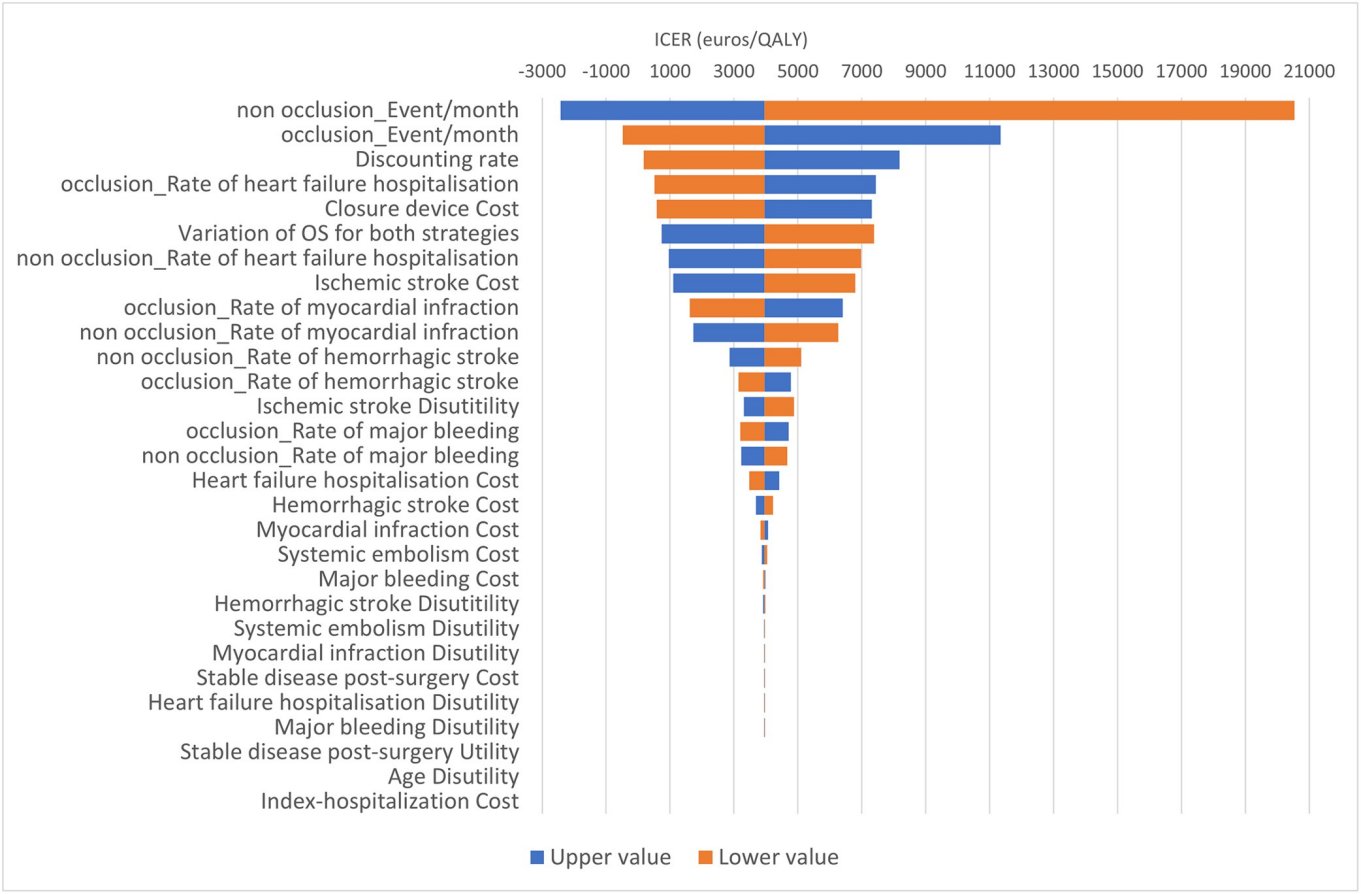
One-way sensitivity analyses: Tornado diagram. Effect of parameter variation on the incremental cost-utility ratio in the scenario analysis assumed 100% medical device. Orange bars represents the lower bound, blue bars represents the upper bounds. *OS*, *overall survival*.

In this scenario, one-way sensitivity analyses (Fig 7) indicated the most influential parameters on the ICUR were also event rate per month (IS and SE) in the “non occlusion” and “occlusion" groups.

We performed a multivariate sensitivity analysis on the same variables as in the base case.

Fig 5B shows that majority of the replicates are located in the north-east quadrant (63.5%). The replicates also extended to the south-west quadrant (26.3%), the south-east quadrant (6%), and also the north-west quadrant (4.2%).

At a WTP threshold of €30,000/QALY, occlusion had 69.5% of chance of being cost-effective (Fig 6).

In the cost/events avoided analysis, (S5B Fig) the majority of the replicates are located in the northeast quadrant of the CE plane and there are some replicates extend into the southeast quadrant.

## 4. Discussion

Our analyses aimed to evaluate the cost-effectiveness of concomitant LAAOs in patients with atrial fibrillation with the postulate that this technique proved to be effective. Indeed, the benefit of LAAOs has been demonstrated in the LAAOS III RCT published in 2021 which relied on a large population [5].

However, more recent findings based on real-world evidence have reported conflicting results: in an observational study on 1640 patients who underwent cardiac surgery between 2001 and 2018, *Kim et al*. [24] did not find a difference in the risk of stroke or mortality at 3.5 years between the occlusion and non-occlusion groups. They also noted a risk of postoperative bleeding in the occlusion group. However, this study selected younger patients with lower CHA₂DS₂ -VASc scores compared to those in LAAOS III and this, together with difference in study design, may explain the discrepancy in findings.

Further studies are still ongoing to assess the efficacy of LAA occlusion, including two trials with substantial sample sizes. The European LAACS-2 study (Left Atrial Appendage Closure by Surgery-2) [25], initiated in 2019 and expected to include 1500 participants, primarily focuses on the number of participants with a stroke as the primary outcome. Additionally, the American LeAAPS study (Left Atrial Appendage Exclusion for Prophylactic Stroke Reduction Trial) [26], which began in 2023, aims to evaluate the benefit of LAAOs using the Atriclip^™^ device to prevent ischemic stroke or systemic arterial embolism with a sample size of 6,500 participants.

From the economic viewpoint, our study confirmed that LAA closure during cardiac surgery is a dominant strategy since it would save an average of €607 per patient while generating a gain of 0.062 quality-adjusted life years (discounted ICUR of €9,775/QALY gained). To the best of our knowledge, we have reported the first long-term estimates on the cost of LAA occlusion.

The difference in efficacy between the two groups was found to be extremely modest (discounted 0.062 QALY / undiscounted 0.081 QALY), corresponding literally to fewer than one additional month of quality-adjusted life generated over a 20-year period. However, the small gain in terms of QALYs was consistent with the equally modest gain as expressed in terms of events avoided, since the absolute risk reduction was 0.022 event avoided /patient (4.8% vs 7%) on the basis of observed data in LAOOS III and 0.072 after modelling (undiscounted value).

According to data from the LAAOS III study [5], the impact of IS / SE reduction on mortality was not visible, and mortality among participants was 3 to 4 times higher than the risk of embolic events. Indeed, mortality rates were 22.6% in the occlusion arm and 22.5% in the non-occlusion arm, while the event rates of interest were only 4.8% in the occlusion arm and 7.0% in the non-occlusion arm.

Although the absolute risk reduction does not appear substantial, a much higher impact could be foreseen in terms of public health after extrapolation of this benefit to the entire population undergoing cardiac surgery. In 2022, in France, 9,630 patients with a secondary diagnosis of atrial fibrillation underwent cardiac surgery, according to data from the PMSI [27].

Our scenario analysis 1, which assumed the exclusive use of the Atriclip^™^ device during LAAOs, suggested that this technique appears to be cost-effective since it generates an incremental cost-utility ratio (ICUR) of €3,952/QALY gained.

Despite being cost-effective under this scenario, the strategy involving the exclusive use of the Atriclip^™^ inevitably raises the question of the economic positioning of this medical device in relation to conventional techniques, for which our analyses suggest dominance.

Indeed, assuming a comparable efficacy between occlusion techniques, a cost-minimisation analysis would give preference to low-cost conventional techniques.

However, decision-making can be more complex when considering several parameters.

The Atriclip^™^ device has the advantage of being specifically designed for AAG closure, unlike other systems, and might therefore be assumed to be safer. Indeed, complications can occur with closure devices such as sutures and staples. Although they appear to be fairly rare, *Wehbe et al* [28] notably described two fatal complications associated with staple and suture closure in 2018. Another theoretical advantage of using a device such as Atriclip^™^, specially "designed" for this indication, is that it may increase the feasibility of the procedure.

In the absence of robust data to corroborate these two aspects, it was not possible to incorporate, in an economic model such as the one we have proposed, differential parameters between the Atriclip^™^ and other occlusion techniques.

Our study, while presenting original data, has limitations. As is inherent in most economic modelling work, it has been based on a large number of model assumptions described in the methodology section of the article. Nevertheless, these assumptions appear to be plausible, particularly from a medical viewpoint, and have been validated by an expert clinician. Also, employing a lifetime horizon has meant that extrapolation techniques had to be used, both for estimating survival and the predicted number of events.

Despite these limitations, the results of our sensitivity analysis suggest a very high probability that this technique was cost-effective or cost-saving depending on the type of occlusion device used during surgery.

The socio-economic characteristics of the LAAOS study population were not described in the main paper published by Whitlock et al [5]. However, given that the study’s inclusion criteria were not highly selective, we can infer that our findings, which rely on the main clinical paper, also apply to populations with diverse socio-economic characteristics. While no French patients were included, 54% (2577 out of 4811) of the patients were enrolled in European centers, encompassing many countries bordering France. Therefore, these results appear to be applicable to French clinical practice.

More generally, our work made a new contribution to demonstrating the efficiency of these techniques. Our model could be updated as new efficacy studies on surgical occlusion become available.

## 5. Conclusion

This study confirms the LAA closure as a dominant long-term strategy in patients with AF and comorbidities. Future economic studies that differentiate between various surgical techniques would be of interest.

## Supporting information

S1 Fig Mortality in French general population with same mean age and gender mix as patients in LAAOS III. Shape parameter = 0.1219917; Scale parameter = 0.0145694199; time scale years. In S1 Fig the survival of the French general population matched by mean age and gender mix to LAAOs is shown by black circles. A Gompertz model fit to the data (red line) provides a very good but slightly imperfect fit. There are virtually no survivors predicted beyond 32 years. The shape parameter for this model was used to model the all-cause mortality from LAAOs III.
(TIF)

S2 Fig Model of mortality in LAAOS III study occlusion and non-occlusion groups for a French population with same mean age and gender mix as patients in LAAOS III. The black data point represents 77.5% survival at 3.8 years based on the Whitlock LAAOS report. The green line (base case) represents a Gompetz model (Shape parameter = 0.1219917; Scale parameter = 0.052726; time scale years) fit to the LAAOS data; The red and brown lines represent plus and minus 10% of life years gained in the base case.
(TIF)

S3 Fig Reconstructed Kaplan-Meier plots for cumulative incidence of events (stroke or embolism). (TIF)

S4 Fig Extrapolation of bathtub models of cumulative incidence of stroke or embolism events. (TIF)

S5 Fig Represents the distribution of incremental cost-effectiveness ratio. A) Base case analysis illustrated on the incremental cost-effectiveness plane. B) Scenario analysis illustrated on the incremental cost-effectiveness plane. Ellipse represents 95% CI for the 1000 means-centred replicates.
(TIF)

S1 Data Raw data relevant to overall survival and event data. (XLSX)

S1 Table Information criteria scores for models of cumulative incidence of stroke or embolism events. (DOCX)

S1 Appendix (DOCX)

## Abbreviations

AF: Atrial Fibrillation
CHEERS: Consolidated Health Economic Evaluation Reporting Standards
DRG: Diagnosis-related groups
EACTS: European Association for Cardio-Thoracic Surgery
ENC: National Cost Scale / Etude Nationale des Couts
ESC: European Society of Cardiology
ICER: Incremental Cost-Effectiveness Ratio
ICUR: Incremental Cost-Utility Ratio
IS: Ischemic Stroke
LAA: Left Atrial Appendage
LAAO: Left Atrial Appendage Occlusion
OS: Overall Survival
PMSI: Information Systems Medicalization Program/Programme de Médicalisation des Systèmes d’Information
QALY: Quality-Adjusted Life Year
QOL: Quality Of Life
RCT: Randomised Controlled Trial
SA: Scenario Analysis
SE: Systemic Embolism
WTP: willingness-to-pay
