# Supplementary figures and images for: Cost-effectiveness of left atrial appendage occlusion during cardiac surgery in France: An economic evaluation based on the LAAOS III study
Source: PLoS One. 2024 May 9;19(5):e0302517. doi: 10.1371/journal.pone.0302517 (PMC11081221; doi:10.1371/journal.pone.0302517)

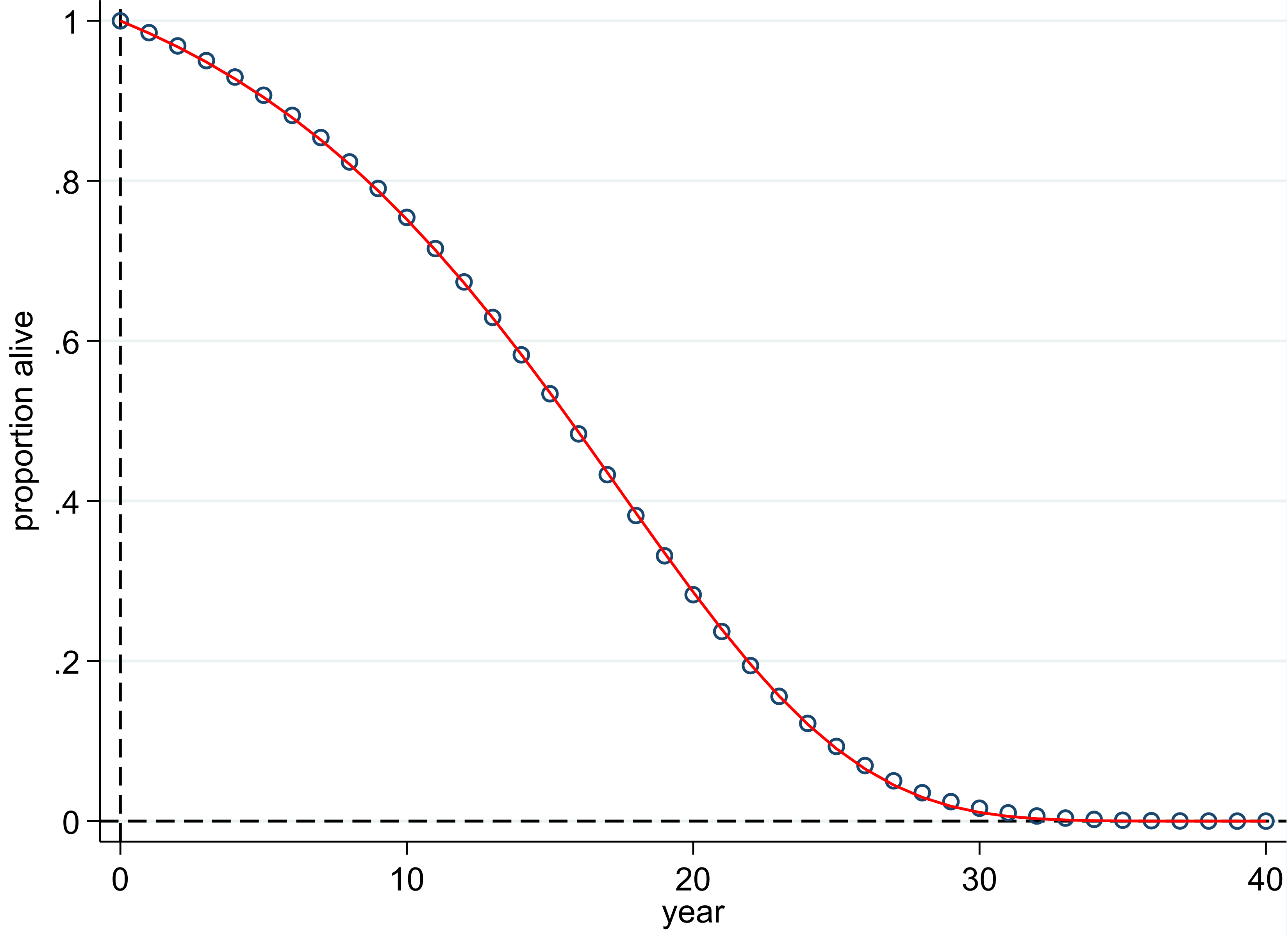

Supplement: S1 Fig — Shape parameter = 0.1219917; Scale parameter = 0.0145694199; time scale years. In S1 Fig the survival of the French general population matched by mean age and gender mix to LAAOs is shown by black circles. A Gompertz model fit to the data (red line) provides a very good but slightly imperfect fit. There are virtually no survivors predicted beyond 32 years. The shape parameter for this model was used to model the all-cause mortality from LAAOs III. (TIF) [file pone.0302517.s001.tif]

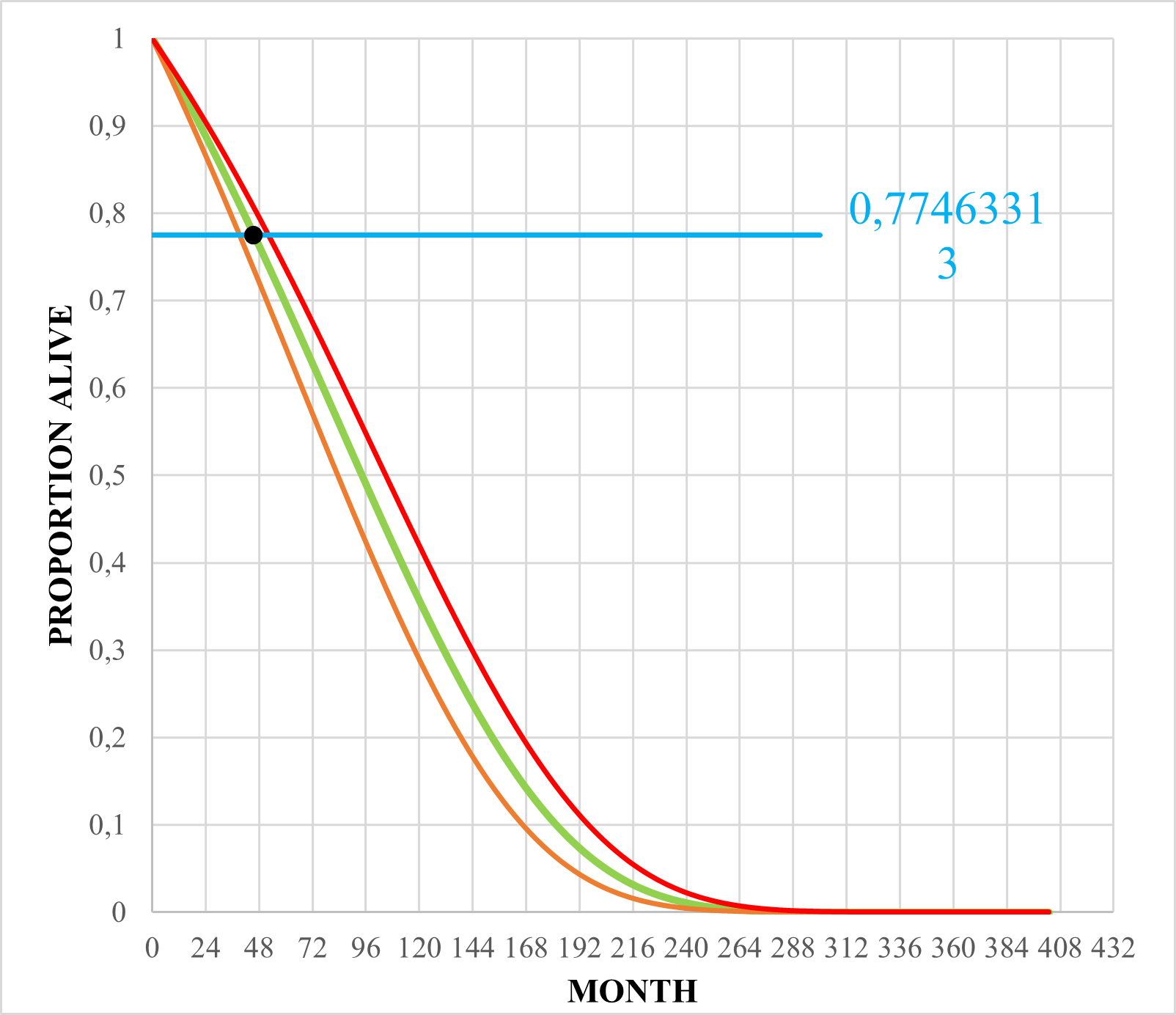

Supplement: S2 Fig — The black data point represents 77.5% survival at 3.8 years based on the Whitlock LAAOS report. The green line (base case) represents a Gompetz model (Shape parameter = 0.1219917; Scale parameter = 0.052726; time scale years) fit to the LAAOS data; The red and brown lines represent plus and minus 10% of life years gained in the base case. (TIF) [file pone.0302517.s002.tif]

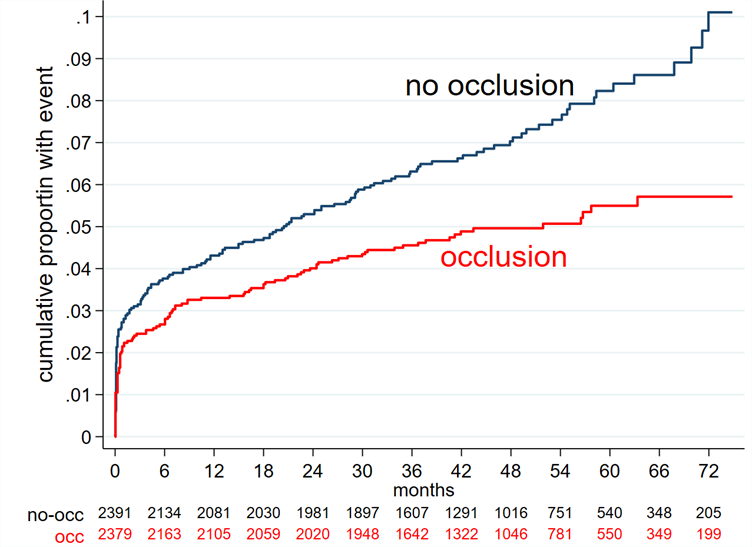

Supplement: S3 Fig — (TIF) [file pone.0302517.s003.tif]

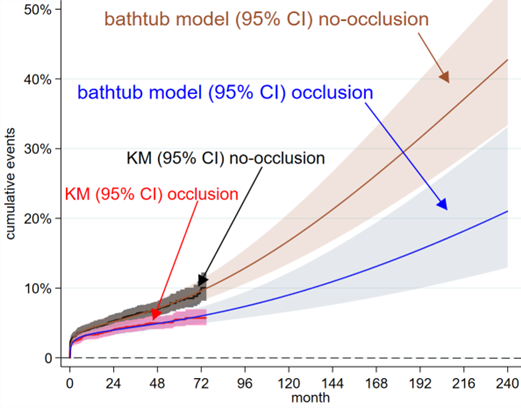

Supplement: S4 Fig — (TIF) [file pone.0302517.s004.tif]

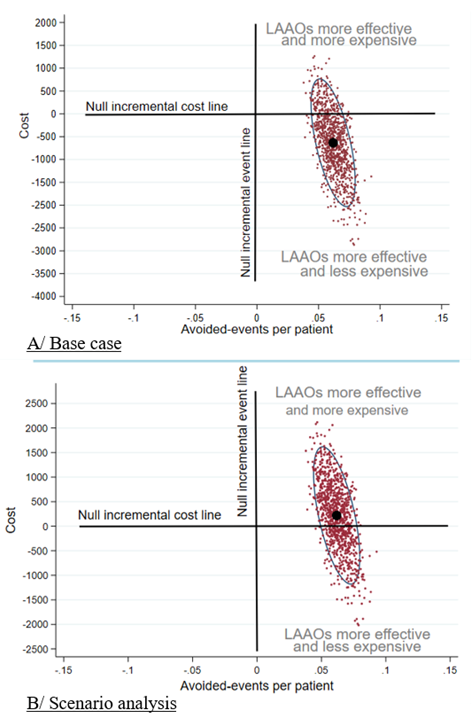

Supplement: S5 Fig — A) Base case analysis illustrated on the incremental cost-effectiveness plane. B) Scenario analysis illustrated on the incremental cost-effectiveness plane. Ellipse represents 95% CI for the 1000 means-centred replicates. (TIF) [file pone.0302517.s005.tif]
