# Supplementary material for: Cost-effectiveness of left atrial appendage occlusion during cardiac surgery in France: An economic evaluation based on the LAAOS III study
Source: PLoS One. 2024 May 9;19(5):e0302517. doi: 10.1371/journal.pone.0302517 (PMC11081221; doi:10.1371/journal.pone.0302517)
Supplement: S1 Appendix — (DOCX) [file pone.0302517.s008.docx]

**Supplementary appendix**

**Figure S1 -** Mortality in French general population with same mean age and gender mix as patients in LAAOS III.

**Figure S2 -** Model of mortality in LAAOS III study occlusion and non-occlusion groups for a French population with same mean age and gender mix as patients in LAAOS III.

**Figure S3** – Reconstructed Kaplan-Meier plots for cumulative incidence of events (stroke or embolism).

**Table S1 -** Information criteria scores for models of cumulative incidence of Stroke or embolism events.

**Figure S4 -** Extrapolation of bathtub models of cumulative incidence of stroke or embolism events.

**Figure S5** – Represents the distribution of incremental cost-effectiveness ratio. A) Base case analysis illustrated on the incremental cost-effectiveness plane. B) Scenario analysis illustrated on the incremental cost-effectiveness plane.

**Figure S1 mortality in French general population with same mean age and gender mix as patients in LAAOS III.** *Shape parameter = 0.1219917; Scale parameter = 0.0145694199; time scale years.*

In Figure S1 the survival of the French general population matched by mean age and gender mix to LAAOs is shown by black circles. A Gompertz model fit to the data (red line) provides a very good but slightly imperfect fit. There are virtually no survivors predicted beyond 32 years. The shape parameter for this model was used to model the all-cause mortality from LAAOs III.

**Figure S2 - Model of mortality in LAAOS III study occlusion and non-occlusion groups for a French population with same mean age and gender mix as patients in LAAOS III.** *The black data point represents 77.5% survival at 3.8 years based on the Whitlock LAAOS report. The green line (base case) represents a Gompetz model (Shape parameter = 0.1219917; Scale parameter = 0.052726; time scale years) fit to the LAAOS data; The red and brown lines represent plus and minus 10% of life years gained in the base case.*


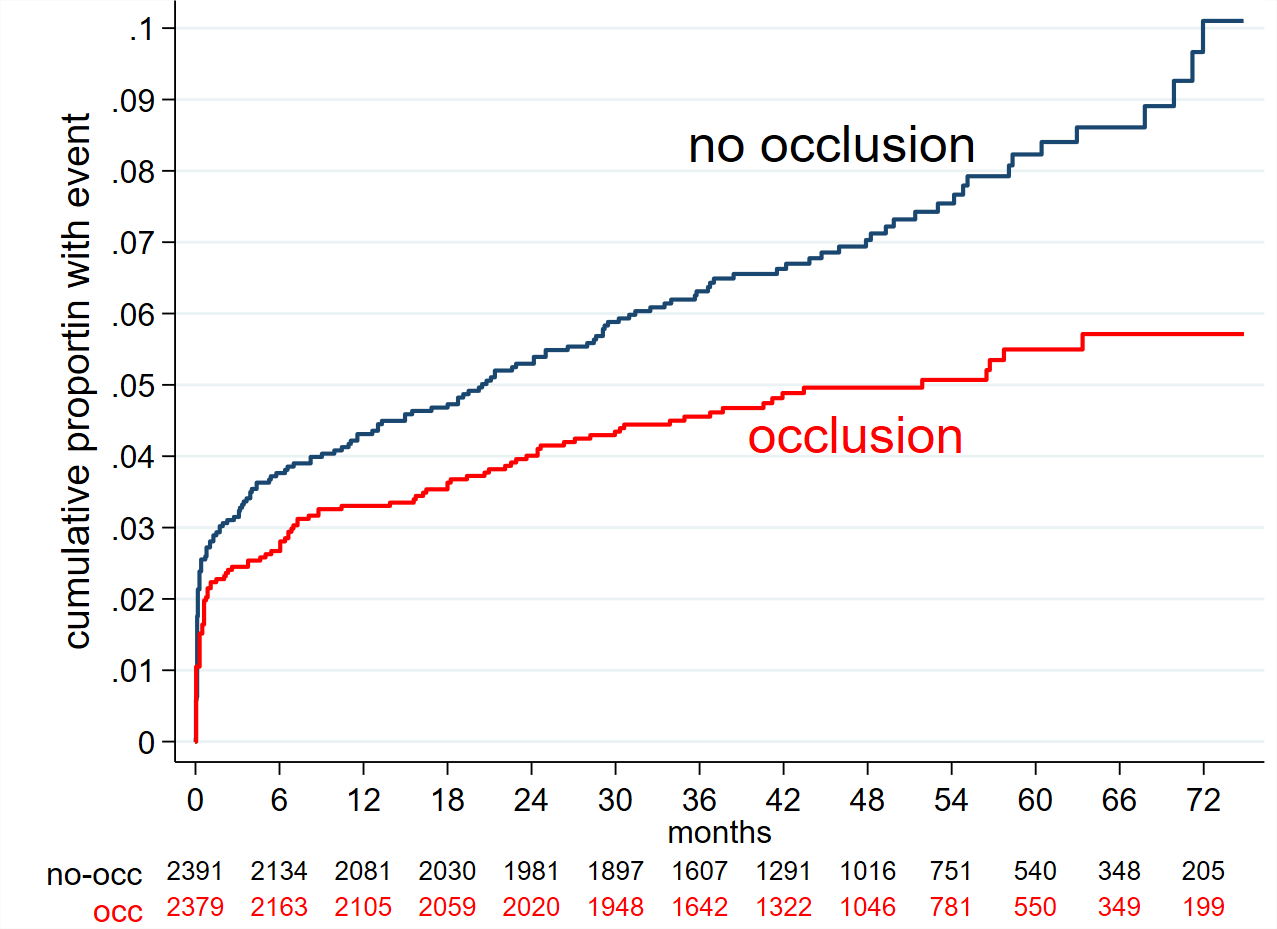


**Figure S3** – Reconstructed Kaplan-Meier plots for cumulative incidence of events (stroke or embolism).

**Table S1 Information criteria scores for models of cumulative incidence of Stroke or embolism events.**

| *number*  *of model*  *parameters* |  | OCC | OCC | *NO OCC* | *NO OCC* | OCC | OCC | *NO OCC* | *NO OCC* |
| --- | --- | --- | --- | --- | --- | --- | --- | --- | --- |
|  | Model | AIC | BIC | *AIC* | *BIC* | AIC+BIC | Rank | *AIC+BIC* | *Rank* |
|  | g-gamma | ****** | | *1,907* | *1,924* | **** | | *3,832* | *5* |
| *1* | exponential | 1,651 | *2,199* | *2,199* | *2,205* | 3,308 | 5 | *4,404* | *6* |
| *2* | Weibull | 1,378 | *1,905* | *1,905* | *1,917* | 2,768 | 2 | *3,822* | *4* |
| *2* | Gompertz | 1,542 | *2,117* | *2,117* | *2,129* | 3,096 | 6 | *4,246* | *7* |
| *2* | lognormal | 1,401 | *1,901* | *1,901* | *1,912* | 2,813 | 4 | *3,813* | *2* |
| *2* | loglogistic | 1,378 | *1,905* | *1,905* | *1,917* | 2,768 | 3 | *3,822* | *3* |
| *2* | Rayleigh | 1,653 | 1,665 | **** | | *3,318* | 7 | **** | |
| *1* | Rayleigh | 2,266 | 2,272 | *3,007* | *3,013* | 4,538 | 8 | *6,020* | *8* |
| *3* | bathtub | 1,375 | 1,392 | *1,851* | *1,869* | 2,767 | 1 | *3,720* | *1* |

** model did not converge.

*
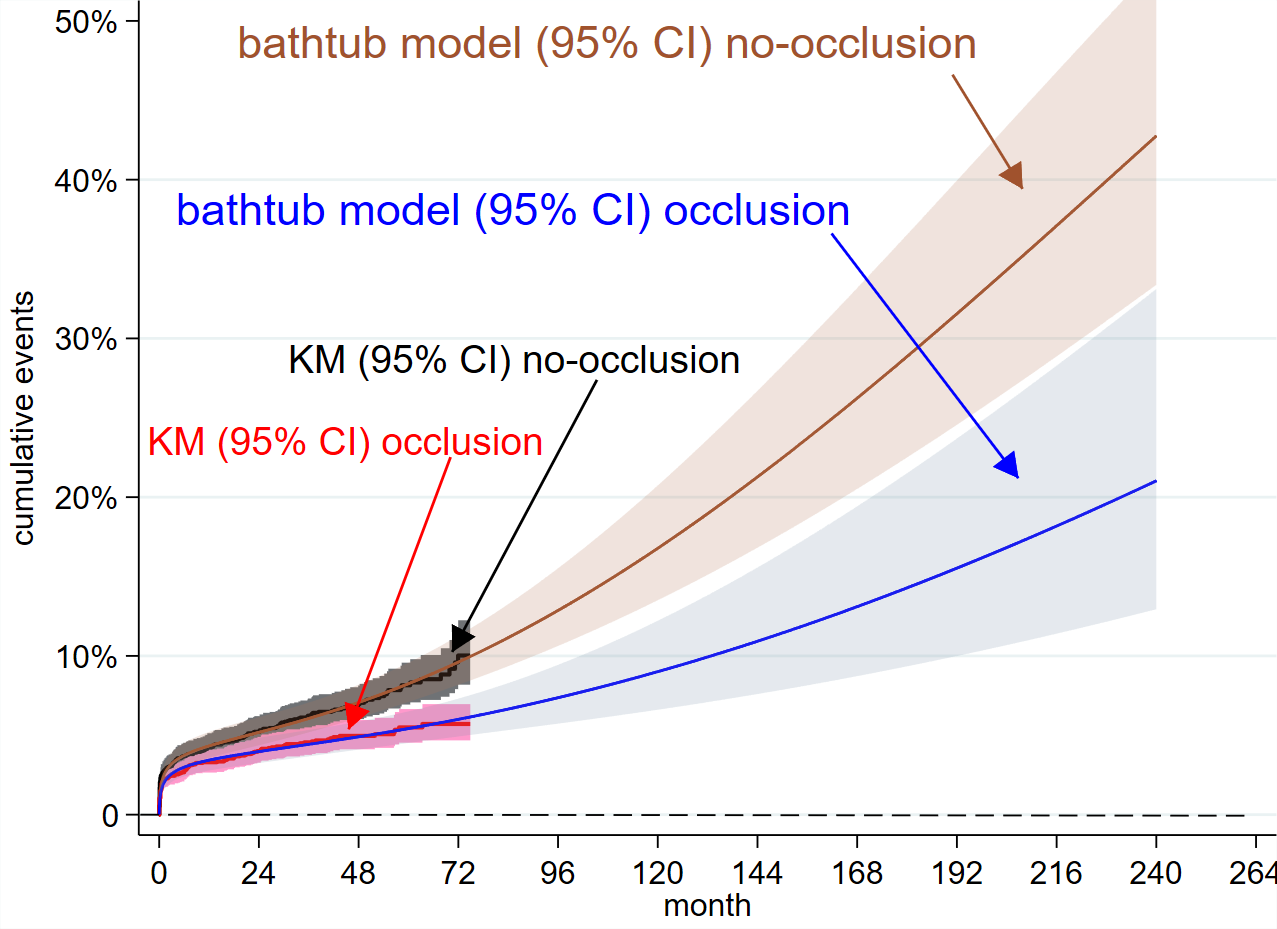
*

**Figure S4 - Extrapolation of bathtub models of cumulative incidence of stroke or embolism events.**


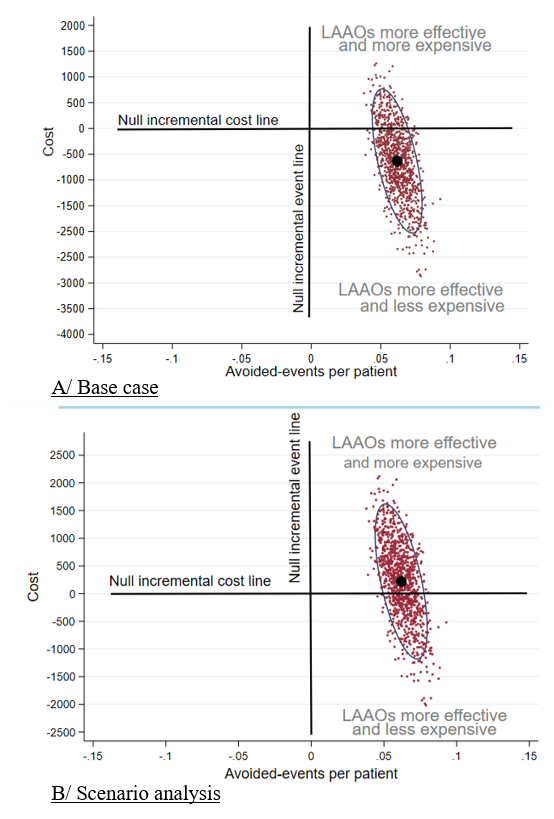


**Figure S5** – Represents the distribution of incremental cost-effectiveness ratio. A) Base case analysis illustrated on the incremental cost-effectiveness plane. B) Scenario analysis illustrated on the incremental cost-effectiveness plane.

Ellipse represents 95% CI for the 1000 means-centred replicates.
